# Longitudinal gene and protein expression changes associated with Mini‐Mental State Examination (MMSE) score in Hispanic/Latino population

**DOI:** 10.1002/alz.086446

**Published:** 2025-01-09

**Authors:** Rashedeh Roshani, Wanying Zhu, Elizabeth Frankel, Hung‐Hsin Chen, Miryoung Lee, Joseph B McCormick, Susan P Fisher‐Hoch, Heather M Highland, Kari E North, Jennifer Elizabeth Below

**Affiliations:** ^1^ Vanderbilt University Medical Center, Nashville, TN USA; ^2^ Academia Sinica, Nangang, Taipei Taiwan; ^3^ The University of Texas Health Science Center at Houston, Houston, TX USA; ^4^ University of North Carolina, Chapel Hill, NC USA; ^5^ Vanderbilt Genetics Institute and Division of Genetic Medicine, Vanderbilt University Medical Center, Nashville, TN USA

## Abstract

**Background:**

Studies suggest that approximately 60 to 70 percent of the variability observed in cognitive abilities during aging can be attributed to genetic factors. Therefore, investigating the longitudinal multiomics changes associated with alterations in cognitive measures, such as the Mini‐Mental State Examination (MMSE) score, is of utmost importance.

**Method:**

Longitudinal changes in gene and protein expression related to MMSE scores were examined in the Cameron County Hispanic Cohort (CCHC), an extensively phenotyped, randomly‐recruited, cohort of Mexican Americans residing at the US‐Mexico border. This study focused on 763 CCHC participants with transcriptomics data and 240 participants with proteomics data. We utilized linear mixed‐effect regression models to estimate the effects of MMSE scores on gene and protein expression, incorporating up to 1044 and 415 measures, respectively. The models were adjusted for covariates such as age, sex, level of education, anxiety score, screening measure of depression (CESD) score, cell type proportions (for transcriptomics analysis only), 3 Principal Components (PC), and 10 Probabilistic Estimation of Expression Residuals (PEER) factors.

**Result:**

Our preliminary results identified 834 genes and 192 proteins that exhibited differential expression based on nominal significance. After mapping the significant proteins to gene IDs, one of the common genes found between the two studies is SCG3, which has previously been associated with cognitive performance and general cognitive ability in the GWAS catalog.

**Conclusion:**

Our results provide evidence of the functional effect driving those GWAS signals, including mapping the effector gene.

